# Apiculture sector policies - positive and negative elements to support healthy market conditions

**DOI:** 10.12688/openreseurope.21244.1

**Published:** 2025-10-07

**Authors:** Henning Lyngsø FOGED, Fatjon HOXHA, Juraj MAJTAN

**Affiliations:** 1 Organe Institute, Skødstrup, DK, 8541, Denmark; 2 Department of Agri-food Technology, Agricultural University of Tirana, Tirana, Albania, 1029, Albania; 3 Institute of Molecular Biology,Slovak Academy of Sciences, Bratislava, Slovakia, Slovakia

**Keywords:** Apiculture, policies, market health, honey

## Abstract

**Background:**

Honey market health indicators, such as production, consumption, and trade balance, exhibit notable variation across countries. Given that policy objectives typically aim to regulate markets either directly or indirectly, it was found pertinent to examine whether correlations can be established between honey market health metrics and policies perceived as beneficial or detrimental to the apiculture sector.

**Method:**

An open, international, web-based survey was designed to gather opinions on policies perceived as positively or negatively influencing the health of the honey market, based on the premise that a healthy honey market is fundamental for a thriving apiculture sector. The survey, created using Google Forms, included only one mandatory question, namely the respondents’ country, and allowed participants to list up to three examples each of positive and negative policies. Members of the BeSafeBeeHoney project, COST Action 22105, were encouraged to assist in gathering responses, ideally from beekeepers’ associations within their countries, though all responses were welcome.

**Results:**

Sixteen survey responses containing 72 policy examples were collected from 13 countries: Albania, Bulgaria, Bosnia and Herzegovina, Croatia, Denmark, Germany, Kosovo, Italy, Serbia, Spain, Slovakia, Türkiye, and the United Kingdom. Of these, one response was gathered via interview. The policy example responses were compiled and organized in a spreadsheet for further grouping and sorting using various criteria. The 13 countries were categorised into three groups based on positive, slightly negative, or negative honey trade balances.

**Conclusions:**

A quantitative analysis of survey responses did not establish a connection between honey trade balances and national policies. Qualitative analyses reflected a general perception of three decisive policy areas, namely i) subsidisation, ii) product classification and labelling, and iii) regulations governing pesticides and veterinarian medicine. Overall, our research highlights challenges within the sector resulting from irregular market mechanisms, with indications that the predominant market channels operate largely beyond public oversight, contributing to concerns about effective regulation and control.

## Background

Honeybees are essential pollinators, with about 75% of major crops relying on pollination, which boosts global crop production by approximately 9% ^1^. The demand for beehive products, especially honey, is increasing across Europe. This trend is largely attributable to the presence of bioactive compounds and the associated health benefits found in many of these products, including notable anti-inflammatory, antioxidant, and antimicrobial properties.

The apiculture sector within the European Union is currently facing several significant challenges ^2^. The total number of beehives declined by 1.2% from 2022 to 2023, primarily due to a reduction in the number of professional producers managing more than 150 hives. Between 2018 and 2022, the average market price for honey decreased from €6.42 to €6.25 per kilogram, largely attributed to increased competition from imported honey of questionable quality ^2^. Notably, this decline occurred during a period marked by a general 42% increase in overall food prices ^3^. Imported honey averages just €1.89 per kilogram, and with imports accounting for approximately 37% of EU consumption, the region remains significantly short of self-sufficiency ^2^. Nearly 76% of honey imports originate from Ukraine and China ^2^.

Concerns about the quality of imported honey are linked to widespread allegations of fraud, particularly adulteration - often involving the blending of honey with inexpensive syrups - which is facilitated by natural variance in honey's constituents under the EU Honey Directive ^4^. Misleading labelling can cause consumers to believe they are purchasing local honey, undermining fair competition and distorting market dynamics. It is estimated that up to 10% of internationally traded honey is adulterated, with rates potentially reaching 30% for imports from certain countries, including China ^5^. The European agriculture umbrella organization COPA-COGECA, which also represents beekeeping associations, has described the state of the honey market as alarming ^6^.

A healthy market is typically defined by self-sufficiency (production plus imports minus exports greater than or equal to consumption, adjusted for change in storages), which is indicated by a positive trade balance, as well as a profitable and stable production economy where sales prices exceed production costs and the price volatility is minimal ^3^. Table 1 presents examples of honey trade balances for selected countries.

**Table 1.** Honey trade balance in 2023 for selected countries, based on Faostat data on export and import as well as population ^9^ and compared with Eurostat based data for EU Member States ^10^. Countries are sorted descending after the Faostat-based trade balance. No statistics exists for Kosovo.

	Faostat data				Eurostat data
	Exports, tonnes	Imports, tonnes	Population, 1,000	Per capita trade balance, kg	Per capita trade balance, kg
Bulgaria	10,991	3,226	6,796	1.14	-0.19
Serbia (incl. Montenegro)	1,510	377	6,773	0.17	
Türkiye	9,386	15	87,271	0.11	
Albania	8	50	2,812	-0.02	
Slovakia	1,694	1,814	5,518	-0.02	-0.17
Spain	27,352	31,005	47,912	-0.08	-0.18
Bosnia and Herzegovina	7	469	3,185	-0.15	
Denmark	2,826	4,154	5,948	-0.22	-0.28
Italy	5,730	24,361	59,499	-0.31	-0.08
Croatia	739	2,775	3,896	-0.52	-0.19
Germany	18,021	64,469	84,548	-0.55	-0.43
UK	2,268	50,917	68,683	-0.71	

**Figure 1.**
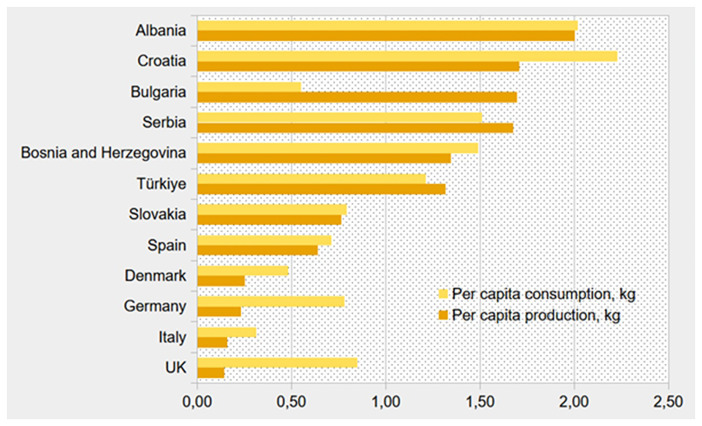
Per capita annual production (dark orange) and consumption (light orange) (kg) of honey in selected BeSafeBeeHoney countries (Based on data of Faostat, 2023 ^9^, production figures for Italy based on Faostat data for 2022 ^9^).

Variations between the reported trade balances, whether derived from Eurostat or Faostat data, suggest that official statistics on the honey market may not be entirely consistent. Also, some discrepancies to honey statistics provided by World Integrated Trade Solutions are observed ^7^. Another indication for this is that Denmark in accordance with information provided by the Danish Beekeeper Association's website ^8^ produces 3–5,000 tonnes of honey annually, which is two to three times higher than the figures reported by Faostat – see Figure 1.

Imprecise statistics regarding honey may result from the fact that a significant portion is produced by hobbyists and sold informally outside of regulated market channels. According to information provided by the Danish Beekeepers Association ^8^, approximately 55% of the honey production is attributed to hobby beekeeping, and similar trends are likely present in other countries. Such circumstances can significantly impede the effectiveness of standard market mechanisms and may create opportunities conducive to unlawful activities.

The BeSafeBeeHoney ^11, 12^ project is a COST Action that receives financial support from the European Union. It operates through international collaboration among researchers and various beekeeping stakeholders to improve the European honey sector, focusing on nutrition, health, abiotic and biotic stressors, and pollination within agrarian ecosystems. The project also includes market analysis and translates technical findings from its different components into policy recommendations.

Behind the general honey market situation in the EU are wide variations between countries, suggesting that the apiculture sector enjoys better market conditions and more favourable policies in some countries than others, since in their essence, policies are made for regulation of markets, directly or indirectly.

These contrasts are already indicated in Table 1, and further illustrated by Figure 1, showing significant differences in consumption and production of honey in selected countries.

Figure 1 presents a general pattern, though with some exceptions, where countries with high honey production also tend to have higher consumption. A comparison of Table 1 and Figure 1 indicates furthermore a possible relationship between a high honey production and a positive trade balance, as seen in Bulgaria, and Serbia. Conversely, countries with lower honey production, such as Germany, Italy, and the UK, are among those with the most negative trade balances. 

The trade balance serves as a key indicator of the health of the honey market and, consequently, the thriving of the apiculture sector. Export and import figures are typically sourced from official trade statistics and are therefore considered more reliable than production and consumption data, notwithstanding discrepancies between Faostat and Eurostat figures.

On basis of Table 1, we can divide countries in 3 groups dependent on their annual trade balance per capita according to Faostat:

1. Positive trade balance: Bulgaria, Serbia, Türkiye
2. Slightly negative trade balance, between 0 and -0.25 kg honey per capita per year: Albania, Slovakia, Spain, Bosnia and Herzegovina, Denmark
3. Negative trade balance, under -0.25 kg honey per capita per year: Italy, Croatia, Germany, UK

Policies that directly influence markets comprise, for instance, trade agreements, subsidy programmes and competition policy. Examples of indirect policies are regulations on feedstuffs, wages, energy, fertilisers, climate and environment. A clear link between policies and markets is not least the case for the European Union, which is concerned about the functioning of the internal market - see for instance Bahr (2024) ^13^.

Direct EU regulation of the honey market happens via the Honey Directive ^4^, latest amended in 2024 by the so-called Breakfast Directive ^14^, which among other includes a definition of honey and requirements to its labelling. EU has enabled support to the beekeeping sector via the Common Agricultural Policy since 1997, whereas it has been mandatory for Member States to prioritise financial support to the sector since 2023 ^15^.

Typically, EU Member States as well as countries outside the EU, have a beekeeping law or a similar regulation, which has the role of recognising the sector and set some basic frames for its activities.

The objective of this research is to evaluate whether the observed market variations can be attributed to differences in policies, including legal frameworks within the countries under consideration, as well as budgetary allocations for sector support.

## Online survey

Policies are basically aimed at regulating markets. On this basis, this article builds on opinions about policies that are either beneficial or detrimental for a thriving apiculture sector, and these policies are compared to some indicators for the health of the apiculture sector in that country.

Opinions about examples of promoting or deteriorating policies were collected via an online survey. In addition, an interview was made and selected literature also taken into consideration.

The online survey was made via Google Forms ^16^. The survey comprised an introduction, where the purpose was explained, it was reminded what the meaning of policies are, and reference were made to prevailing GDPR rules and possibilities for clarification ^17^. The introduction part made it possible to leave the name, e-mail address and organisational affiliation of the respondent, and it included the only obligatory field of the entire survey, namely what country the respondent was based in. Following that, there were three similar sections, where the respondent could write (freely) about a policy that was considered to promote the apiculture sector, and three sections, where the respondent could indicate policies that were considered to deteriorate the apiculture sector. It was possible on a voluntary basis to provide details, such as to indicate the type of the policy, provide a link to the specific policy, give a reference of the policy (law number or alike.), indicate the geographical scope, and indicate the impact of the policy (impact groups being aligned with the themes of the BeSafeBeeHoney work groups). None of the questions were compulsory, except as already mentioned, the country of the respondent.

Using Google forms means that the respondent could have an automated language translation of the survey, most accessible in case it was opened in a Chrome browser. Using Google Forms also means that management of the survey was facilitated by functions to register and edit survey questions, receive email notifications every time a response was collected, view responses in a compiled format as well as individually, and download responses in comma separated file (.csv) format for further analyses.

The survey was launched at the first BeSafeBeeHoney conference, held in Larissa in Greece in the days 28–29 May 2024, where it was promoted in connection to the conference session on policies, and via a poster. A QR code was displayed at the poster and in a PowerPoint for introduction to the WG5 session of the conference, to make access to the survey as easy as possible. In addition, the survey was promoted via a newsletter, send to about 130 Work Group 5 members on 8 July and again on 13 September 2024.

## Quantitative analysis of responses

**Figure 2.**
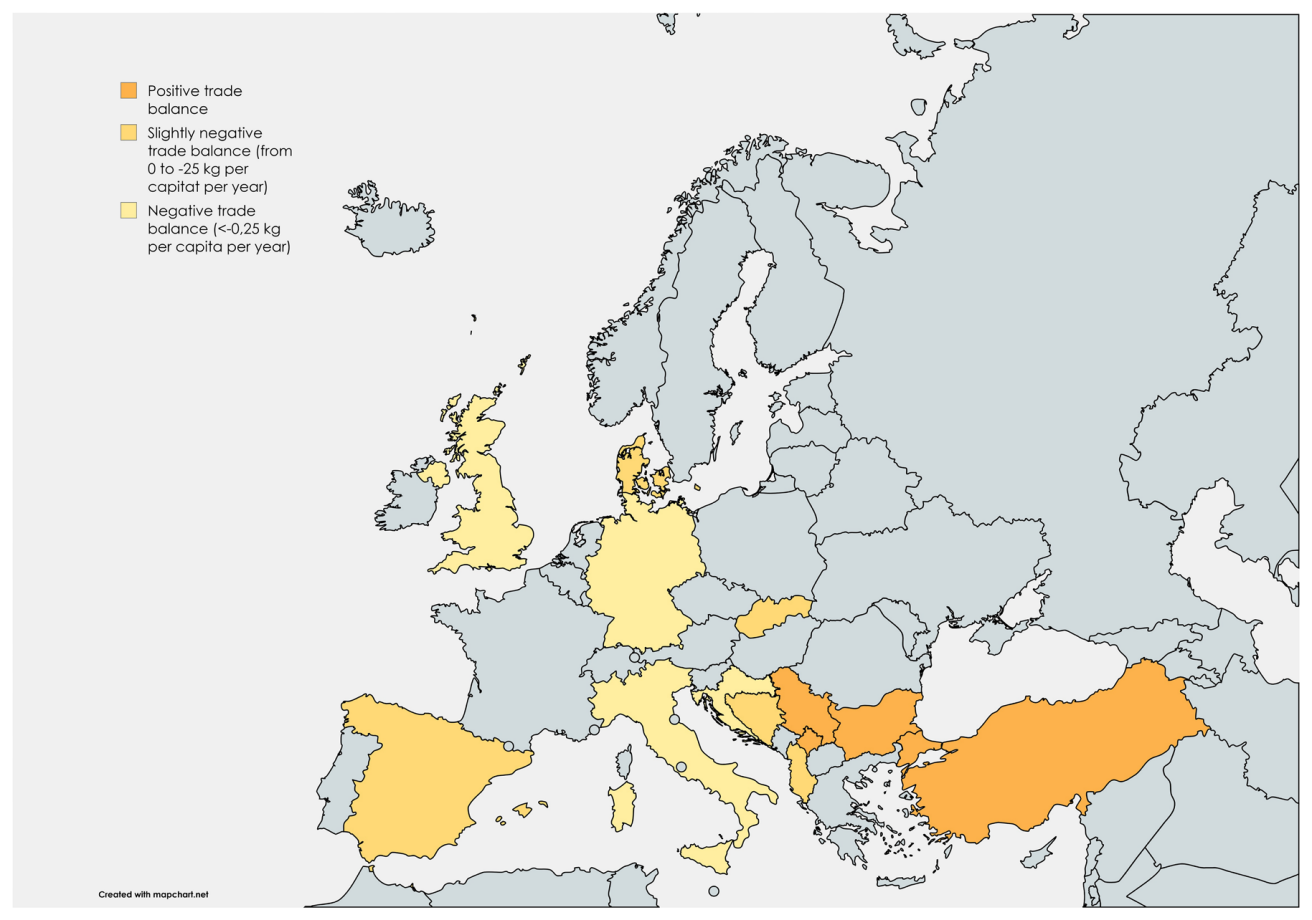
Countries represented in responses to the policy survey, coloured according to the size of their honey trade balance: Dark orange – Positive trade balance, medium orange – slightly negative trade balance (from 0 to -0.25 kg per capita per year, light orange – negative trade balance (<-0.25 kg per capita per year).

15 answers were collected from respondents representing 12 European countries, and an interview-based response was additionally collected in one country. The countries are shown in Figure 2, using the above definition of positive, slightly negative or negative honey trade balance. 

For two countries, the response was empty or unclear, whereas three countries (Serbia, Bosnia & Herzegovina and Türkiye) each provided two responses. Thus, 72 policy examples were provided by 16 respondents from 13 countries ^1^, and Table 2 shows, how these are distributed over issues and perceived positive or negative impact.

**Table 2.** Survey responses, distributed over issues and perceived positive of negative impact.

Issue	Number of responses		
	Promotional policies	Deteriorating policies	Total
Abiotic stressors	4	8	12
Biotic stressors	3	2	5
Disease	8	1	9
Nutrition	7	7	14
Production systems & economy	24	8	32
In total	46	26	72

Table 2 shows that more than 40% of responses relate to the issue of production systems and economy, whereas policies on abiotic and biotic stressors, disease and nutrition are seen as having less importance for a thriving apiculture sector. It can also be concluded that each respondent on average provided 4.5 examples of promotional or deteriorating policies, and that almost two thirds of the provided policy examples were perceived as having positive impact for the apiculture sector.

Using Figure 2’s division of responding countries, Table 3 shows the division on number of promotional and deteriorating policy examples, collected with the survey. 

**Table 3.** The number of promoting and deteriorating national policy examples for countries with positive, slightly negative and negative trade balance for honey, with averages per responding country shown in brackets.

	Number of responses (number of responses per responding country)		Comments
	Promotional policies	Deteriorating policies	
Positive	11 (5.5)	3 (1.5)	Three countries: Türkiye and Serbia - no national policies mentioned for Bulgaria
Slightly negative	11 (2.2)	6 (1.2)	Five countries: Bosnia & Herzegovina, Slovakia, Denmark, Spain and Albania
Negative	6 (3.0)	2 (1.0)	Four countries: Croatia and United Kingdom – Germany and Italy not mentioning national policies

33 policy examples were related to EU or other international policies.

A correlation between policies and trade balances would indicate a consistent pattern in which countries with negative trade balances reported more deteriorating policies, while those with positive trade balances would give more examples of promotional policies. However, Table 3’s quantitative analysis does not show any evidence of a relationship between annual trade balance per capita and the number of promotional or deteriorating policies. 

## Qualitative analysis of responses

Since policy examples were collected using open questions, there are no identical responses, but there are responses that relate to the same issues:

•    
**Abiotic stressors**
      Positive examples relate to EU policies, dealing with public support to information spreading (education, seminars), regulations that reduce the use of pesticides to a minimum. A Codex for Bee Products in Türkiye reduce the risk for chemicals to enter honey and other bee products.
      Negative examples were given for non-EU countries, such as UK, where pesticides that were prohibited when UK was EU member, now are allowed again (e.g. neonicotinoids). For Serbia was mentioned non-restricted use and sale of antibiotics, that are used for plant disease treatment.
•    
**Biotic stressors**
      As beneficial policy example was mentioned a national policy on the control/elimination of Asian Hornets (Vespa veluntina), meaning that a rapid identification and location of attacks lead to appropriate disposal of Asian Hornets' nests and prevent or limit the spread of the Asian hornet (and associated detrimental effects on bees) across the UK.
•    
**Disease control**
      Bosnia & Herzegovina and Croatia mentioned as example of positive policies that they have veterinarian legislation, that also cover measures to prevent, control and survey bee disease.
      Of negative examples were from Danish side mentioned a private scheme to make combatting of the Varroa mite more efficient, but the frustration is that the scheme is ineffective since beekeepers cannot be sanctioned if they do not follow the scheme. Spain also mentions a parallel example of this policy defect.
•    
**Nutrition**
      Some positive policy examples provided are not entirely clear since they refer to the Honey Directive, but it is not concerned about bee nutrition. Some examples see EU’s regulatory framework in support of biodiversity as positive, as well as EU’s Soil Mission, that would mean more healthy foraging possibilities for bees.
      Of negative policy examples were for Bosnia & Herzegovina mentioned that inspection of honey is not anymore being a responsibility of veterinarian authorities. Another example pointed at the insufficiency of EU’s food regulations, since they alone concern about lead contamination of honey.
•    
**Production systems and economy**
      Several examples of positive policies dealt with beekeepers’ access to subsidisation, for instance a subsidy per beehive in Serbia, an EU regulation making Member States’ subsidies of the apiculture sector mandatory, the possibility to use EU’s CAP funding for additional support to grow fallow fields with flowers in Denmark, and support for bee queen purchase in Serbia.
      Other positive examples mentioned the possibility for beekeepers to sell honey directly to consumers without formal business registration, EU’s quality schemes to protect products under different Designations of Origins, EU’s amendment of the Honey Directive to require the country of origin to be labelled on the honey, and beekeepers’ possibility to rent out beehives to crop producers and in this way generate income from pollination services.
      Türkiye mentioned some beneficial policies, including a broad regulation aiming to ensure the sustainability of beekeeping by determining the principles regarding all kinds of production, breeding, accommodation, obtaining breeding material, fixed and migratory beekeeping, taking the necessary measures regarding the transportation of bee colonies, standardization of tools, machines and materials, development of honey plants agriculture, and training. The regulation covers issues related to project planning, queen bee breeding, artificial insemination of honeybees and registration of honeybee colonies.
      Negative responses were mainly expressing a frustration of the lack of policies to avoid a suspected adulteration, specifically of imported honey. They also included a response that points at the lack of policies to counteract a demography of ageing beekeepers.

It is emphasised that the above points are not attempting to provide a complete overview of response, but alone to present some response examples.

To some extent, the survey responses overlap with policy issues that were captured by Foged (2024) from keynote speeches at the BeSafeBeeHoney conference in Larissa in May 2024 ^3^. The issues listed in the referenced policy brief can be seen as identification of missing or failing policies dealing with the following:

- Ineffective measures to combat the Varroa mite – it was suggested to increase work for more genetic resistant bees and introduce more tight regulation on bee trade, including bee queen trade to avoid contamination.
- Insufficient regulation of use of pesticides and other chemicals – it was suggested to work for harmonising of the regulations for registration, trade and use of pesticides within all the EU to avoid farmers buying pesticides in neighbour countries that are illegal in their own country.
- Fraud and adulteration – it was recommended to work for better and more diverse labelling, including labelling for honeys’ analysed content of nutraceutical constituents, stronger border control, and to introduce labelling that includes more detailed traceability information.

## Conclusions and discussion

A quantitative analysis of 39 examples of policies that were associated with individual countries did not identify any correlation between a country's honey trade balance, measured as kilograms of honey per citizen per year, and the quality of its apiculture sector related policies.

Qualitative analysis of the survey responses, including 72 examples of policies, highlights three key policy issues perceived as most decisive for the quality of the honey market and its ability to sustain a thriving apiculture sector:

- First, subsidisation schemes are considered crucial for supporting the sector, with respondents noting that such policies can prioritise objectives including education and information dissemination, development initiatives, disease eradication programmes, and investment in beehives. Subsidisation is generally viewed positively, with explicit calls for increased funding. The expansion and mandatory financial support for the apiculture sector within the EU is widely regarded as beneficial.
- Second, there is a strong belief to the importance of enhanced product classification systems and accurate labelling, utilising reliable methods to verify label information with honey quality standards. These measures are considered necessary to combat fraud, promote fair competition, distinguish high-quality honey products rich in nutraceutical components, and reassure consumers about the absence of chemical residues. While this issue garners significant attention, effective laboratory methods for fraud detection have yet to be identified, while traceability requirements may offer more feasible solutions to combat fraud.
- Third, concerns persist regarding overly permissive regulations governing the approval, registration, and use of veterinary medicines and pesticides, which may negatively impact the sector. Although EU regulations are generally regarded as superior to those outside the EU, there remains a need for harmonised regulations across EU Member States.

The policy survey provides valuable insights into apiculture sector policies regarded as either beneficial or detrimental to a sustainable honey market and the ongoing development of apiculture. Additionally, it highlights challenges within the sector resulting from irregular market mechanisms, with indications that the predominant market channels operate largely beyond public oversight, contributing to concerns about effective regulation and control. 

The policy survey was spread via BeSafeBeeHoney project participants, mainly being researchers without primary concerns for policies, and being researchers having an inherent caution about sharing subjective opinions. The BeSafeBeeHoney project participants were for this reason encouraged to be helpful to obtain responses from beekeepers and especially beekeepers associations in their countries, since these are familiar with policies. At least two responses were given by representatives of beekeepers’ associations, which is good, and also that a few other responses were provided by researchers, who are also beekeepers. Some of the responses were of limited quality and not sufficiently clear to include in the survey analysis.

Ideally, responses would have been obtained from beekeepers’ associations in all of the more than 40 BeSafeBeeHoney countries; however, this was not achieved, and it is important to acknowledge the challenges of eliciting participation in online surveys. Nevertheless, given these constraints, we believe the survey responses are reasonably representative and comprehensive, supporting the validity of our conclusions for consideration in the European apiculture policy process.

## Ethical approval

Ethical approval and consent were not required.

## Data Availability

Foged, H. L., Hoxha, F., & Majtan, J. (2025). Template and responses of policy survey. In Apiculture sector policies - positive and negative elements to support healthy market conditions (25 August 2025). Zenodo. https://doi.org/10.5281/zenodo.16941550
