# Apiculture sector policies - positive and negative elements to support healthy market conditions

**DOI:** 10.12688/openreseurope.21244.2

**Published:** 2026-01-30

**Authors:** Henning Lyngsø FOGED, Fatjon HOXHA, Juraj MAJTAN

**Affiliations:** 1 Organe Institute, Skødstrup, DK, 8541, Denmark; 2 Department of Agri-food Technology, Agricultural University of Tirana, Tirana, Albania, 1029, Albania; 3 Institute of Molecular Biology,Slovak Academy of Sciences, Bratislava, Slovakia, Slovakia

**Keywords:** Apiculture, policies, market health, honey

## Abstract

**Background:**

The apiculture sector is facing severe challenges, and the honey market health is generally low. Whereas some countries have a positive honey trade balance, the self-sufficiency with honey in the EU is a low as 63% and there are suspicion of fraud with cheap honey imports, deteriorating the business economy like a vicious circle. Given that policies typically aim to regulate markets either directly or indirectly, it was found pertinent to identify examples of policies that are perceived as either being beneficial or deteriorating for a thriving apiculture sector, for the purpose of establishing a basis for sharing these examples via policy recommendations.

**Method:**

An international, web-based survey using open-ended questions was designed to gather opinions on policies perceived as positively or negatively influencing the health of the honey market. The questionnaire allowed respondents to list up to three examples each of positive and negative policies. Members of the COST Action 22105 BeSafeBeeHoney project, were encouraged to assist in gathering responses.

**Results:**

Seventeen responses containing 74 policy examples were collected from 13 countries: Albania, Bulgaria, Bosnia and Herzegovina, Croatia, Denmark, Germany, Kosovo, Italy, Serbia, Spain, Slovakia, Türkiye, and the United Kingdom. The provided policy examples were compiled and organised in a spreadsheet for further grouping and sorting using various criteria. The 13 countries were categorised into three groups based on positive, slightly negative, or negative honey trade balances.

**Conclusions:**

Qualitative analyses for patterns in responses reflected a general perception of three decisive policy areas, namely i) subsidisation, ii) product classification and labelling, and iii) regulations governing pesticides and veterinary medicine. Both positive and deteriorating policy examples were given for these issues. However, our research highlights fundamental challenges within the sector resulting from irregular market mechanisms, with indications that the predominant market actors operate largely beyond public oversight, contributing to concerns about effective regulation and control.

## Background

Honeybees are essential pollinators, with about 75% of major crops relying on pollination, which boosts global crop production by approximately 9% ^1^. The demand for beehive products, especially honey, is increasing across Europe. This trend is largely attributable to the presence of bioactive compounds and the associated health benefits found in many of these products, including notable anti-inflammatory, antioxidant, and antimicrobial properties.

However, the apiculture sector within the European Union is currently facing several significant challenges. The total number of beehives declined by 1.2% from 2022 to 2023, primarily due to a reduction in the number of professional producers managing more than 150 hives. Between 2018 and 2022, the average market price for honey decreased from €6.42 to €6.25 per kilogram, largely attributed to increased competition from imported honey of questionable quality ^2^. Notably, this decline occurred during a period marked by a general 42% increase in overall food prices ^3^. Imported honey averages just €1.89 per kilogram, and with imports accounting for approximately 37% of EU consumption, the region remains significantly short of self-sufficiency ^2^. Nearly 76% of honey imports originate from Ukraine and China ^2^.

Concerns about the quality of imported honey are linked to widespread allegations of fraud, particularly adulteration, often involving the blending of honey with inexpensive syrups, which is facilitated by natural variance in honey's constituents under the EU Honey Directive ^4^. It is estimated that up to 10% of internationally traded honey is adulterated, with rates potentially reaching 30% for imports from certain countries, including China ^5, 6^. The European agriculture umbrella organisation COPA-COGECA, which also represents beekeeping associations, has described the state of the honey market as alarming ^7^.

A healthy market is typically defined by the degree of self-sufficiency (the size of the production in relation to the size of the consumption), which is closely related to and often being used as a synonym for the trade balance (exports minus imports), as well as a profitable and stable production economy where sales prices exceed production costs and the price volatility is minimal ^3^. Both the degree of self-sufficiency and the trade balance serve as key indicators of the health of the honey market and, consequently, the thriving of the apiculture sector. Export and import figures are typically sourced from official trade statistics and are therefore considered more reliable than production and consumption data, meaning trade-balances considered more reliable indicators than self-sufficiencies.

Behind the general honey market situation in the EU, there are wide variations between countries. For instance, whereas EU’s average self-sufficiency with honey as aforementioned is as low as 63% (100% minus imports of 37%), a few EU countries like Romania, Czechia, Portugal and Hungary are more than self-sufficient ^8^. The differences in countries honey trade balances may be attributed to the size of the honey production, including availability of floral resources ^9^ and other factors, as well as the honey consumption, that depends on culinary traditions and many other factors.

However, the trade balance, including the size of the honey production is also expected to be affected by apiculture sector related policies, since in their essence, policies are made for regulation of markets, directly or indirectly. A clear link between policies and markets is not least the case for the European Union, which is concerned about the functioning of the internal market - see for instance Bahr (2024) ^10^. Policies that directly influence markets comprise, for instance, trade agreements, subsidy programmes and competition policy. Examples of indirect policies are regulations on feedstuffs, wages, energy, fertilisers, climate and environment.

Direct EU regulation of the honey market happens via the Honey Directive ^4^, latest amended in 2024 by the so-called Breakfast Directive ^11^, which among other includes a definition of honey and requirements to its labelling. EU has enabled support to the beekeeping sector via the Common Agricultural Policy (CAP) since 1997, whereas it has been mandatory for Member States to prioritise financial support to the sector since 2023 ^12^. Typically, EU Member States as well as countries outside the EU, have a beekeeping law or a similar regulation, which has the role of recognising the sector and set some basic frames for its activities. However, regulation of the apiculture sector, including harmonising national policies with EU directives in EU Member States is implemented differently into national policy frameworks. For EU it is given the aforementioned market challenges clear, that the policy framework is not fully effective in supporting the apiculture sector in all Member States.

The BeSafeBeeHoney ^13, 14^ project is a COST Action supported by the COST Association - European Cooperation in Science and Technology, that receives financial support from the European Union. The project focuses on improving the safety, quality, and sustainability of beekeeping and bee products in Europe. It operates through international collaboration among researchers and various beekeeping stakeholders to improve the European honey sector, focusing on nutrition, health, abiotic and biotic stressors, and pollination within agrarian ecosystems. In addition, an important and transversal objective of the project is to develop and disseminate recommendations for policies and practices that are beneficial for the apiculture sector, and vice-versa work for removal of policies that impairs the sector.

On this background, the objective of this research is to collect examples among BeSafeBeeHoney partner countries of policies that are perceived as beneficial or deteriorating for a thriving apiculture sector. The wider purpose is to establish a basis for development of BeSafeBeeHoney policy recommendations, and thus work for a wider implementation of beneficial apiculture sector policies, and vice-versa discontinuation of detrimental policies. The research builds on the assumption that the wider aim of apiculture sector policies is to support healthy markets, and that honey trade balances is the best indicator for that, wherefore collected policy examples are compared to the market health of the associated countries, notwithstanding that several other factors also affect market health.

The current research aims at contributing to existing research of apiculture sector policies, and it fills a gap by collecting and analysing perceptions on current policies that are perceived as either beneficial or deteriorating for the honey market, for the purpose of a more disseminated use of beneficial, and discontinuation of deteriorating apiculture sector policies. For instance, Maderson (2023) ^15^ analysed obstacles and opportunities for co-production of agricultural policy with beekeepers, but did not focus on specific policy issues. Bertoni *et al.* (2025) ^16^ analysed 29 variables, considered drivers for the performance of beekeeping activities across the environmental, social, and economic dimensions, thus contributing to the understanding of factors of importance for beekeepers’ optimisation of their honey production, however, without clearly relating the findings to current apiculture sector policies in a wider geographical context. Delso *et al.* (2019) ^17^ explains how EU’s (then planned) Common Agricultural Policy (CAP) for 2021-27 can support the apiculture sector, and the work can be interpreted as a guidance to the BeeLife network for ways to impose their influence on the (then future) national CAP strategic plans via policy processes to ensure these are being formulated to give the best direct and indirect support to the sector, whereas the current research aims at assessing more widely the already implemented policies that are perceived as either beneficial or detrimental. Bixby *et al.* (2023) ^18^ analysed factors of importance for optimising honey production and its profitability, including loss factors, and while this gives some insight into ways for improving honey self-sufficiencies, the research does not relate to specific polices. Bruneau *et al.* (2024) ^19^ are analysing the honey market, among others, on basis of Eurostat and Faostat data, mentioning challenges that include fraud, and provide some policy recommendations that represent the authors’ perspectives, being employers or members of BeeLife, while the current research more widely collects and analyses perceptions about already implemented policies.

## Method for collection of perceptions

Opinions about examples of promoting or deteriorating policies were collected via an online survey questionnaire. In addition, an interview was made in one case.

The online survey was performed using Google Forms ^20^. The survey questionnaire ^21^ comprised an introduction, where the purpose was explained, it was reminded what the meaning of policies are, and reference were made to prevailing GDPR rules ^22^ and possibilities for clarifications. The introduction part made it possible to leave the name, e-mail address and organisational affiliation of the respondent, and it included the only obligatory field of the entire questionnaire, namely the country of the respondent. Following that, there were three similar sections with entirely open-ended questions, where respondents could write freely about a policy considered beneficial for the apiculture sector, and three sections, where respondents could indicate policies considered detrimental to the sector. It was possible, on a voluntary basis to provide details, such as to indicate the type of the policy, provide a link to the specific policy, provide a reference to the policy (law number or alike), indicate the geographical scope, and indicate the impact of the policy, impact groups being aligned with the themes of the BeSafeBeeHoney work groups.

The survey was exploratory, encouraging participation from individuals that willingly would express their opinion about beekeeping policies, including beekeepers and their associations, using the BeSafeBeeHoney network for promoting the survey. The responses were not intended to be statistically generalisable, but rather for obtaining insight into examples of apiculture sector related policies across diverse national contexts inside and outside EU, that were perceived as notably beneficial or deteriorating for a thriving apiculture sector.

The use of open-ended questions was well considered and takes basis in the situation that honey policies are structured widely different in national policy frameworks, and that it adds further to the complexity that BeSafeBeeHoney members represents both EU and non-EU member countries. Given this context, using questionnaires with closed-ending questions, largely giving respondents the possibility to answer “Yes” or “No” to a defined set of questions, would have meant an inherent risk for not having included questions on issues that in a given country would be considered of being among the most supportive or the most detrimental for the apiculture sector. The use of open-ended questions is a well proven social science method, especially for the aims of exploring issues related to perceptions ^23^, and is a well-suited methodology in the case of co-creation purposes.

The decision to use open-ended questions is entirely in line with the objective and means that responses must be given a qualitative analysis for identification of patterns in respondents’ perceptions. Open-ended questions are not likely to yield responses using similar formulations, and cannot be analysed statistically. Each expressed perception is unique and has a value in itself.

Using Google Forms meant that the respondent could have an automated language translation of the survey, most accessible in case it was accessed in a Chrome browser. Using Google Forms also meant that management of the survey was facilitated by functions to register and edit survey questions, receive email notifications every time a response was collected, view responses in a compiled format as well as individually, and download responses in comma separated file (.csv) format for further analysis.

The survey was launched at the first BeSafeBeeHoney Conference, held in Larissa in Greece in the days 28–29 May 2024, where it was promoted in connection to the conference session on policies, and via a poster. A QR code was displayed on the poster and in a PowerPoint for the introduction to the WG5 session of the Conference, to ensure easy access to the survey questionnaire. In addition, the survey was promoted via a Newsletter, send to more than 130 Work Group 5 members on 8 July and again on 13 September 2024. Additionally, the policy survey was promoted through the project webpage ^14^ and the project newsletter, The BeeLetter, in June 2024. 

Any numerical summaries and country groupings are provided solely to describe the dataset and should not be interpreted as evidence of causal or statistical relationships.

## Description of collected policy examples

16 responses were collected, representing 12 European countries, and an interview-based response was additionally collected in one country.

One response from Poland was empty, probably due to some technical issue, whereas four countries (Serbia, Bosnia & Herzegovina, Croatia and Türkiye) each provided two responses. Thus, 74 policy examples were provided by 17 respondents from 13 countries ^1^. The responded policy examples are organised in a spreadsheet ^21^ to ease analysing. Table 1 shows, how the provided policy examples are distributed over issues and perceived as having positive or negative impact on honey market health.

**Table 1.** Survey responses, distributed over issues and perceived as having positive of negative impact on honey market health.

Issue	Number of responses		
	Promotional policies	Deteriorating policies	Total
Abiotic stressors	4	8	12
Biotic stressors	3	2	5
Disease	8	1	9
Nutrition	7	7	14
Production systems & economy	26	8	34
In total	48	26	74

The responses represent countries with a total of approximately 230,000 tonnes of annual honey production, whereof 83,500 tonnes in EU Member States, based on Faostat data ^24^. For comparison, the total EU honey production was 286,000 tonnes in 2022 ^2^.

Table 1 shows that 34 of 74, or around 46% of expressed perceptions relates to the issue of production systems and economy, which compared to issues on abiotic and biotic stressors, disease and nutrition are seen as having the highest importance for a thriving apiculture sector. It can also be concluded that each respondent (excluding Poland) on average provided 4.4 examples of promotional or deteriorating policies, and that 48 of 74, or almost two thirds of the provided policy examples were perceived as having positive impact for the apiculture sector.

Table 2 presents honey trade balances for countries represented among the survey responses.

**Table 2.** Honey trade balance in 2023 for selected countries, based on Faostat data on export and import as well as population ^24, 27^ and compared with Eurostat based data for EU Member States ^28^. Countries are sorted descending after the Faostat-based trade balance. No statistics exists for Kosovo.

	Faostat-based data				Eurostat data
	Exports, tonnes	Imports, tonnes	Population, 1,000	Per capita trade balance, kg	Per capita trade balance, kg
Bulgaria	10,991	3,226	6,796	1.14	-0.19
Serbia	1,510	377	6,773	0.17	
Türkiye	9,316	15	87,271	0.11	
Slovakia	1,803	1,762	5,518	0.01	-0.17
Albania	4	51	2,402	-0.02	
Spain	27,811	31,383	47,912	-0.07	-0.18
Bosnia and Herzegovina	7	469	3,185	-0.15	
Denmark	2,349	4,186	5,948	-0.31	-0.28
Italy	5,730	24,361	59,499	-0.31	-0.08
Croatia	733	2,776	3,896	-0.52	-0.19
Germany	18,574	64,769	84,548	-0.55	-0.43
UK	2,274	50,927	68,683	-0.71	

**Figure 1.**
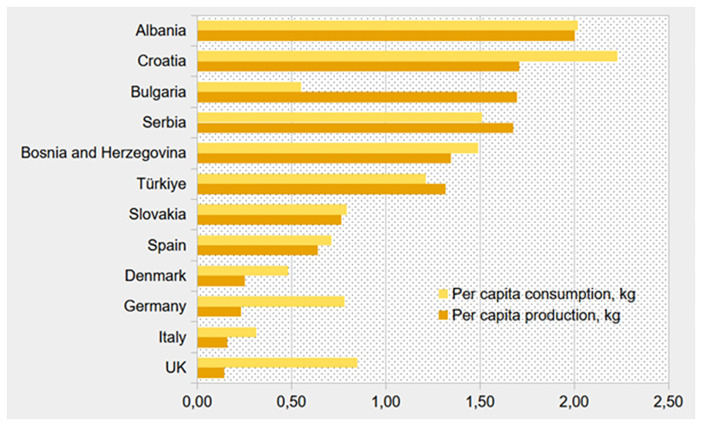
Per capita annual production (dark orange) and consumption (light orange) (kg) of honey in selected BeSafeBeeHoney countries (Based on data of Faostat, 2023 ^24, 27^, production figures for Italy based on Faostat data for 2022 ^24, 27^).

As a side effect on the attempt to describe the survey dataset by statistics on the represented countries, there were discovered some discrepancies. Variations between the reported trade balances, whether derived from Eurostat or Faostat data, suggest that official statistics on the honey market may not be entirely consistent. Also, some deviation to honey statistics provided by World Integrated Trade Solutions are observed ^25^. Another indication for this is that Denmark in accordance with information provided by the Danish Beekeeper Association's website ^26^ produces 3–5,000 tonnes of honey annually, which is two to three times higher than indicated by the Faostat-based figures behind Figure 1.

Imprecise statistics regarding honey may result from the fact that a significant portion is produced by hobbyists and sold informally outside of regulated market channels. According to information provided by the Danish Beekeepers Association ^26^, approximately 55% of the honey production is attributed to hobby beekeeping, and similar trends are likely present in other countries. Such circumstances can significantly impede the effectiveness of standard market mechanisms and may create opportunities conductive to unlawful activities.

These contrasts are already indicated in Table 2, and further illustrated by Figure 1, showing significant differences in consumption and production of honey in countries behind the responses.

Figure 1 presents a general pattern, though with some exceptions, where countries with high honey production also tend to have higher consumption. A comparison of Table 2 and Figure 1 indicates furthermore a possible relationship between a high honey production and a positive trade balance, as seen in Bulgaria and Serbia. Conversely, countries with lower honey production, such as Germany, Italy, and the UK, are among those with the most negative trade balances.

On basis of Table 2, we can divide countries in 3 groups dependent on their annual trade balance per capita according to Faostat:

1. Positive per capita trade balance: Bulgaria, Serbia, Türkiye and Slovakia
2. Slightly negative trade balance, between 0 and -0.5 kg honey per capita per year: Albania, Spain, Bosnia and Herzegovina, Denmark and Italy
3. Negative trade balance, under -0.5 kg honey per capita per year: Croatia, Germany and UK

**Figure 2.**
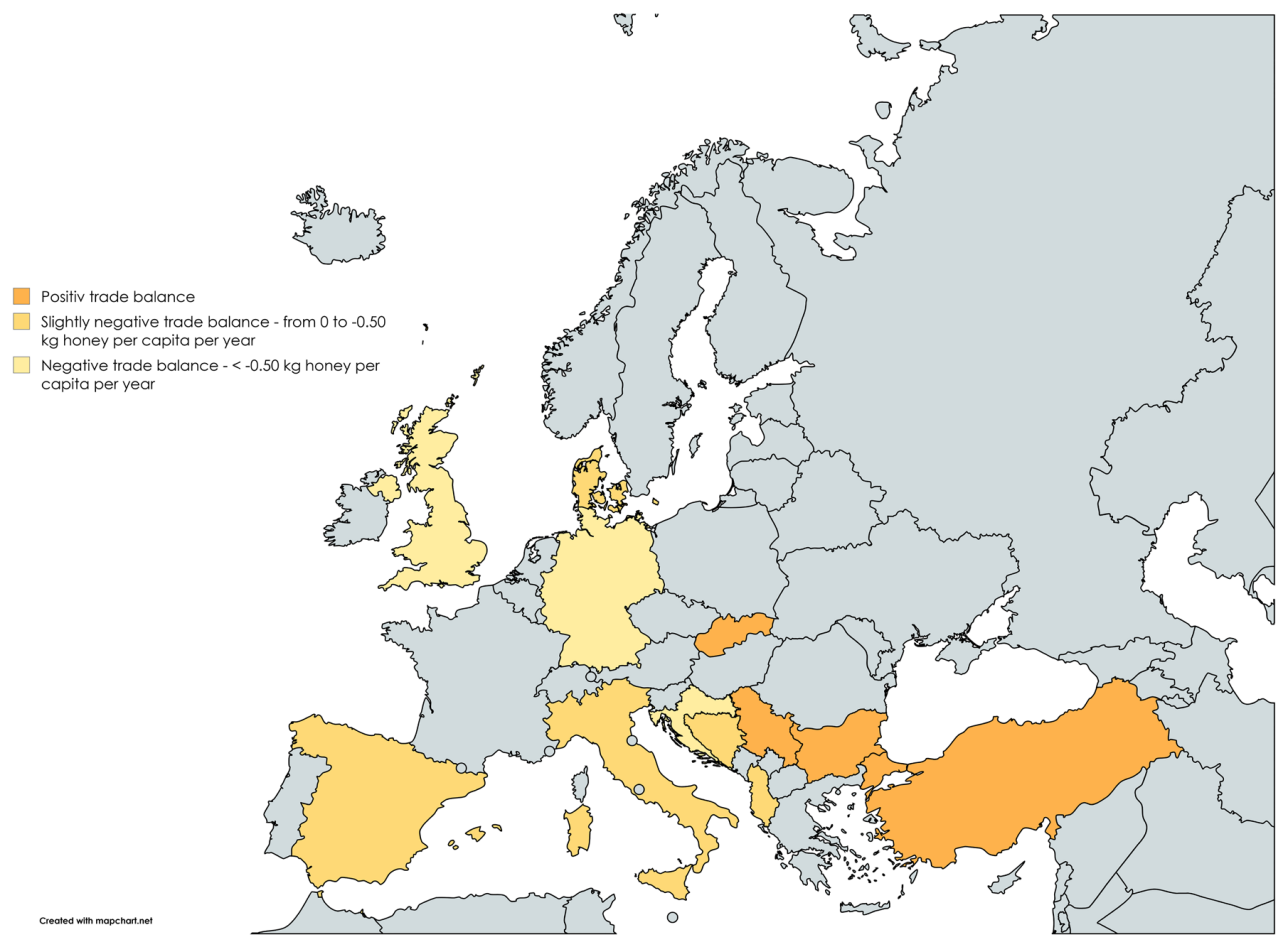
Countries represented in responses to the policy survey, coloured according to the size of their honey trade balance: Dark orange – Positive trade balance, medium orange – slightly negative trade balance (from 0 to -0.50 kg per capita per year, light orange – negative trade balance (<-0.50 kg per capita per year).

The countries are shown in Figure 2, using the above definition of positive, slightly negative or negative honey trade balance.

Table 3 shows how 39 out of 74 national policy examples divides on promotional and deteriorating examples for the three trade balance groups of countries, whereas 35 policy examples were related to EU or other international policies.

**Table 3.** The number of promoting and deteriorating national policy examples for countries with positive, slightly negative and negative trade balance for honey, with averages per responding country shown in brackets.

	Number of responses (number of responses per responding country)		Comments
	Promotional policies	Deteriorating policies	
Positive	12 (4.0)	3 (1.0)	Four countries: Serbia, Türkiye, Slovakia - no national policies mentioned for Bulgaria
Slightly negative	10 (2.5)	6 (1.5)	Five countries: Bosnia & Herzegovina, Denmark, Spain and Albania - Italy not mentioning national policies
Negative	6 (3.0)	2 (1.0)	Three countries: Croatia and United Kingdom – Germany not mentioning national policies

## Qualitative analysis of responses

Since policy examples were collected using open-ended questions, there are no identical responses, but there are responses that relate to the same issues and some general patterns in responses are identified:

- **Abiotic stressors**
  Abiotic stressors, including pesticides and veterinary medicine was among the policy areas that respondents saw as most decisive for a thriving apiculture sector. The issue was exemplified in five deteriorating policies, including an example from UK, where pesticides that were prohibited when UK was EU member, now are allowed again (e.g. neonicotinoids), and an example from Serbia that mentions non-restricted use and sale of antibiotics. Four beneficial policy examples were given, including one that relate to EU regulations that reduce the use of pesticides to a minimum, and one dealing with a Codex for Bee Products in Türkiye that reduce the risk for chemicals to enter honey and other bee products.
  In line with this, a key recommendation at the 2024 BeSafeBeeHoney Conference was to work for harmonising of the regulations for registration, trade and use of pesticides within all the EU to avoid farmers buying pesticides in neighbour countries that are illegal in their own country ^29^.
- **Biotic stressors**
  As beneficial policy example was mentioned a national policy on the control/elimination of Asian Hornets (Vespa veluntina), meaning that a rapid identification and location of attacks lead to appropriate disposal of Asian Hornets' nests and prevent or limit the spread of the Asian hornet (and associated detrimental effects on bees) across the UK.
  Bosnia & Herzegovina as well as Croatia mentioned as example of positive policies that they have veterinarian legislation, that also cover measures to prevent, control and survey bee disease.
- **Disease control**
  Of negative examples were from Danish side mentioned a private scheme to make combatting of the Varroa mite more efficient, but the frustration is that the scheme is ineffective since beekeepers cannot be sanctioned if they do not follow the scheme. Spain also mentions a parallel example of this policy defect.
  A key recommendation given on this issue at the 2024 BeSafeBeeHoney conference was to increase work for more genetic resistant bees and introduce more tight regulation on bee trade, including bee queen trade to avoid contamination ^29^.
- **Nutrition**
  Some positive policy examples provided are not entirely clear since they refer to the Honey Directive, which is not concerned about bee nutrition. Some examples see EU’s regulatory framework in support of biodiversity as positive, as well as EU’s Soil Mission, that would mean more healthy foraging possibilities for bees.
  Of negative policy examples were for Bosnia & Herzegovina mentioned that inspection of honey is not any more being a responsibility of veterinarian authorities. Another example pointed at the insufficiency of EU’s food regulations, since they alone concern lead contamination of honey, but not regulates other similar contaminants.
- **Production systems and economy**
  No less than 13 examples of positive policies dealt with various form of financial support, for instance a subsidy per beehive in Serbia, an EU regulation making Member States’ subsidies of the apiculture sector mandatory, the possibility to use EU’s CAP funding for additional support to grow fallow fields with flowers in Denmark, and support for bee queen purchase in Serbia. In addition, one response regretted that subsidies are not available for beekeeping in cities in Croatia, and another response regrets that compulsory EU support for the sector does not prioritise measures to counteract a demography of ageing beekeepers. All in all, the issue of public support for the sector, and the way this support is designed is overall seen as the most important policy measure to sustain healthy markets.
  Respondents considered the issues of labelling and classification as the second most important policy area, and saw this both in relation to the ability to combat fraud and to raise the income from sales of specific high honey qualities. Seven positive policy examples were given, for instance praising EU’s quality schemes to protect products under different Designations of Origins, and EU’s amendment of the Honey Directive to require the country of origin to be labelled on the honey. There were also provided four examples of counteracting policies, including regrets that EU’s Honey Directive ^4^ does not provide effective solutions for protecting the EU market and prevent imports of cheap and adulterated honey qualities, and another example pointing at the unlucky rules for labelling of organic food, which confuses consumers to believe the product is local.
  The issue of labelling and traceability was also mentioned at the 2024 BeSafeBeeHoney conference, where it was recommended to work for better and more diverse labelling, including labelling for honeys’ analysed content of nutraceutical constituents, stronger border control, and to introduce labelling that includes more detailed traceability information ^29^.
  Türkiye mentioned some beneficial policies, including a broad regulation aiming to ensure the sustainability of beekeeping by determining the principles regarding all kinds of production, breeding, accommodation, obtaining breeding material, fixed and migratory beekeeping, taking the necessary measures regarding the transportation of bee colonies, standardisation of tools, machines and materials, development of honey plants agriculture, and training. The regulation covers issues related to project planning, queen bee breeding, artificial insemination of honeybees and registration of honeybee colonies.

It is emphasised that the above points are not attempting to provide a complete overview of provided policy examples, but alone to present some policy examples, including patterns of policy examples, i.e. cases of responded policies dealing with the same issue, although formulated differently.

## Conclusions

Qualitative analysis of 74 apiculture sector related policy examples provided on basis of a survey using open-ended questions, highlights three key policy issues perceived as most decisive for the quality of the honey market and its ability to sustain a thriving apiculture sector:

- First, subsidisation schemes are considered crucial for supporting the sector, with respondents noting that such policies can prioritise objectives including education and information dissemination, development initiatives, disease eradication programmes, and investment in beehives. Subsidisation is generally viewed positively, with explicit calls for increased funding. The expansion and mandatory financial support for the apiculture sector within the EU is widely regarded as beneficial. No less than 15 provided policy examples related to the issue of subsidisation and public support to the sector, meaning that this issue was highlighted by almost all respondents.
- Second, evidenced by 11 provided policy examples, there is a strong belief to the importance of enhanced product classification systems and accurate labelling, utilising reliable methods to verify label information with honey quality standards. These measures are considered necessary to combat fraud, promote fair competition, distinguish high-quality honey products rich in nutraceutical components, and reassure consumers about the absence of chemical residues. While this issue garners significant attention, effective laboratory methods for fraud detection have yet to be identified, while traceability requirements may offer more feasible solutions to combat fraud.
- Third, concerns persist regarding overly permissive regulations governing the approval, registration, and use of veterinary medicines and pesticides, which may negatively impact the sector. Although EU regulations are generally regarded as superior to those outside the EU, there remains a need for harmonised regulations across EU Member States. While a total of nine policy examples dealt with this issue, these were characterised by frustration over regulations, that do not prioritise the business interests of the apiculture sector.

The policy survey provides valuable insights into apiculture sector policies regarded as either beneficial or detrimental to a sustainable honey market and the ongoing development of apiculture. Additionally, it highlights challenges within the sector resulting from irregular market mechanisms, with indications that the predominant market actors operate largely beyond public oversight, contributing to concerns about effective regulation and control.

## Discussion

Our findings clarify needs for future research in the following areas:

- Public, financial support to the sector is perceived as having a great importance. However, subsidisation is justified by delivery of services of value for the society, and ways to maximise that within the apiculture sector is yet to be thoroughly investigated, especially in consideration of the large part of the honey production and marketing taking place outside public control. With other words, subsidisation should comply with EU’s conditionality principles ^3^, in order to avoid subsidies having effect as merely social security payments, which may be counterproductive to society targets related to other policy areas, e.g. agri-environment, despite from risking to lower productivity and product quality in the apiculture sector.
  For instance in Albania, the recently adopted Law on Beekeeping No 20/2023 and the national financial direct support of 1,000 Lekë (~ 10€) per hive demonstrate an increasing governmental commitment to the apiculture sector. However, this support scheme is not yet aligned with the structure and conditionality mechanisms of the EU Common Agricultural Policy. Albania is therefore in a transitional phase, gradually adapting its regulatory framework to move closer to EU standards while still operating under a distinct national support model. There is a profound need in a country like Albania to investigate, how financial support via conditionalities would maximise its effect in relation to a thriving apiculture sector.
- Research is needed for finding better ways for labelling and classification of honey to document its quality, prevent adulterated honey from being marketed as native honey, and ensure fair market conditions for beekeepers. Given an inherent difficulty in proving the quality of honey that shows wide variations in its composition and for which there are not identified any precise indicator that can be validated in a laboratory or otherwise, the focus of this research should be given to develop other ways to reach the objectives, including more advanced traceability requirements than already decided.
- The perception is that EU regulations on pesticides and veterinarian medicine is advantageous to that in non-EU countries, but that there is still a need to harmonise it inside EU, avoiding illegal trade between EU Member States. It is a policy research task to identify solutions for a more harmonised pesticide and veterinarian medicine regulation in the EU, without hampering overall food security and competitiveness.
- The efforts to clarify statistics on production, consumption, import and export reflects, as aforementioned, that a large part of the honey production and marketing takes place outside public control. This is a specific charm of the apiculture sector, but it hampers regulation of the sector. Therefore, as part of the suggested investigations to identify the most optimal ways of supporting the sector, it must be considered, how small-size amateur beekeepers can come under public control via subsidisation schemes in smart ways without requiring VAT accounting and the like and without much administration. In this respect, research should identify possibilities for use of state-of-the-art IoT solutions, for instance for identifying and tracking beehives and document their production.

It is emphasised that we find our conclusions as well as the mentioned recommendations for future research areas as valid and well justified by the 74 examples of apiculture related policies collected via the survey using open-ended questions. Our conclusions and recommendations gives a good basis for development of recommendations for shaping of future apiculture related policies. 

Many responses pointed at similar policy issues, and we therefore feel that additional responses would not add substantial details and quality to our findings. We had initially hoped for more responses, but despite a general situation of survey fatigue, we do believe the survey responses are representative and sufficiently comprehensive, supporting the validity of our conclusions for consideration in the European apiculture policy dialogue and research efforts.

## Ethical approval

Ethical approval and consent were not required.

## Data Availability

Foged, H. L., Hoxha, F., & Majtan, J. (2025). Template and responses of policy survey. In Apiculture sector policies - positive and negative elements to support healthy market conditions. Zenodo. https://doi.org/10.5281/zenodo.17826786.

## Footnotes

^1^ *Kosovo is counted as a country in this respect, but neither Eurostat nor Faostat provides honey statistics for Kosovo*.
