# Supplementary material for: Apiculture sector policies - positive and negative elements to support healthy market conditions
Source: Open Res Eur. 2026 Jan 30;5:305. Originally published 2025 Oct 7. [Version 2] doi: 10.12688/openreseurope.21244.2 (PMC13096782; doi:10.12688/openreseurope.21244.2)
Supplement: Peer review history

# Reviewer report: Reviewer response for version 2

**DOI:** 10.21956/openreseurope.23960.r72524

**Published:** 2026-04-20

**Authors:** Antonio de Jesus Cenobio-Galindo

**Affiliations:** 1 Universidad Autónoma del Estado de Hidalgo, Hidalgo, Mexico

The authors adequately addressed the observations made; I have no further comments on this matter.

Is the study design appropriate and does the work have academic merit?

No

Is the work clearly and accurately presented and does it cite the current literature?

No

If applicable, is the statistical analysis and its interpretation appropriate?

No

Are all the source data underlying the results available to ensure full reproducibility?

No

Are the conclusions drawn adequately supported by the results?

No

Are sufficient details of methods and analysis provided to allow replication by others?

No

Reviewer Expertise:

honey, stingless bees, food biotechnnology

I confirm that I have read this submission and believe that I have an appropriate level of expertise to confirm that it is of an acceptable scientific standard.

# Reviewer report: Reviewer response for version 2

**DOI:** 10.21956/openreseurope.23960.r68911

**Published:** 2026-02-25

**Authors:** Małgorzata Dżugan

**Affiliations:** 1 Uniwersytet Rzeszowski, Rzeszów, Poland

I highly appreciate the Authors' efforts to reformulate the manuscript to more clearly describe the study's purpose, provide detailed explanations in the methodological section, and emphasize that the responses collected through open-ended questions are not suitable for statistical analysis but for qualitative analysis, aimed at comparing policies implemented in various countries participating in the BeSafeHoneyBee program. The modification introduced, which involved relating the obtained data to the state (trade balances) of the honey market in the respondents' countries, allowed for a more logical and transparent structure of the manuscript.

 However, one aspect still requires modification, as it introduces unnecessary confusion in the manuscript and is inconsistent with scientific research methodology: the description of collected policy examples. The authors write, "16 responses were collected, representing 12 European countries, and an interview-based response was additionally collected in one country" (which is consistent with Table 2 and Figure 1, which present 12 countries). It is unclear why the authors state that "One response from Poland was empty, probably due to some technical issue." If this was a design error in the Google form (a mandatory field had to be indicated), the form should be rejected and not included in further analysis. Including this empty questionnaire in the dataset results in further errors, for example, in the sentence "Thus, 74 policy examples were provided by 17 respondents from 13 countries," while there were 12 countries (nothing is known about Polish apiculture sector related policies). And further, "It can also be concluded that each respondent (excluding Poland) on average provided 4.4 examples...."

 It is strange that the authors, having data from a Polish respondent (mandatory) and a BeSafeBeeHoney network member, did not bother to obtain data on strategies implemented in Poland.

 Once the Authors have reviewed and modified the appropriate number of survey responses in accordance with the caveats outlined above, I am ready to update the article record to "Approved."

Is the study design appropriate and does the work have academic merit?

Partly

Is the work clearly and accurately presented and does it cite the current literature?

Yes

If applicable, is the statistical analysis and its interpretation appropriate?

No

Are all the source data underlying the results available to ensure full reproducibility?

Partly

Are the conclusions drawn adequately supported by the results?

No

Are sufficient details of methods and analysis provided to allow replication by others?

Yes

Reviewer Expertise:

NA

I confirm that I have read this submission and believe that I have an appropriate level of expertise to confirm that it is of an acceptable scientific standard, however I have significant reservations, as outlined above.

# Response

**Published:** 2026-03-05

**Authors:** Henning Lyngsø FOGED

We appreciate your positive comments on the improvements in the second version of our manuscript.  We regret your confusion over the description of the responses to the policy survey, including the quantification.  Concerning your remarks on a response from Poland: The statement on the response from Poland is part of describing the response we had to the policy survey, which we find relevant.  Concerning your statement: “ the form should be rejected and not included in further analysis. Including this empty questionnaire in the dataset results in further errors, for example, in the ….” It is unfortunately not clear for us, on which basis you assume an empty submission (apart from “Poland”) could be part of the 74 collected policy examples, and we would even see it impossible to categorise some empty fields as being either a positive or negative policy example, or any of the other ways we characterised the collected policy examples? We remind that you also have access to the dataset holding the mentioned 74 responses. We also remind, as stated in the manuscript, that Kosovo is counted as a country (by the respondent, therefore also by us), but not included in figures and tables that are based on statistics, since such are not produced for Kosovo by Eurostat and Faostat. Concerning your questioning why we could not have an answer from Poland, please acknowledge that responding to the policy survey happened on a voluntary basis. We have mentioned in our manuscript that the response to the policy survey regrettably was limited, considering it having been promoted through the BeSafeBeeHoney network, which comprise more than 40 countries. We would have hoped to have more responses, but do on the other hand consider the response being sufficient for drawing solid conclusions, considering the very clear patterns found in the responses. Please also notice that the policy survey did not require respondents to reveal their identity, for very well considered reasons – and as you have noticed, the only obligatory question was the country. Therefore, logically, since “Poland” was the only text in one response, we cannot know who the response came from and we cannot go back to that person and request another response.  Conclusively, we have once again reviewed the manuscript with respect to the description of the responses to the policy survey, including the quantification, and find that all is correct presented. Also, it is not clear for us, on which basis you assume that an empty response could be included in the analysis.  Therefore, on the background of the aforementioned information, and with all respect, kindly approve our manuscript as it is. Otherwise, we would be glad for your clarification of the specific amendments you request of the manuscript, including the quantification of survey responses, and we would of course implement these as far as they are correct.

# Reviewer report: Reviewer response for version 2

**DOI:** 10.21956/openreseurope.23960.r68910

**Published:** 2026-02-11

**Authors:** Ester Noguer-Juncà

**Affiliations:** 1 University of Lleida, Lleida, Spain

The new document is ok.

Is the study design appropriate and does the work have academic merit?

Partly

Is the work clearly and accurately presented and does it cite the current literature?

Yes

If applicable, is the statistical analysis and its interpretation appropriate?

No

Are all the source data underlying the results available to ensure full reproducibility?

Partly

Are the conclusions drawn adequately supported by the results?

Partly

Are sufficient details of methods and analysis provided to allow replication by others?

No

Reviewer Expertise:

food tourism, food marketing, primary sector and tourism, tourism and gender

I confirm that I have read this submission and believe that I have an appropriate level of expertise to confirm that it is of an acceptable scientific standard.

# Reviewer report: Reviewer response for version 1

**DOI:** 10.21956/openreseurope.22978.r64629

**Published:** 2025-12-09

**Authors:** Antonio de Jesus Cenobio-Galindo

**Affiliations:** 1 Universidad Autónoma del Estado de Hidalgo, Hidalgo, Mexico

Following the evaluation of the manuscript, I present below some comments that require attention to consider the work for publication.

 The title presents the study as a policy analysis; however, the document does not demonstrate that the elements are sufficient.

 The abstract states that no differences were found between policies and trade balance; however, the survey design is inadequate to support any assessment, especially regarding representativeness across nations.

  Regarding the previous comment, the research question cannot be adequately answered due to the methodological design.

 The introduction lacks data relevant to the study, such as policies applied to the beekeeping sector or honey market regulations.

 It is understood that the survey used open-ended questions, which prevents standardizing responses, hinders comparisons, and therefore does not allow for quantitative analysis.

 Procedures for ensuring reliability or coding the data are not described.

 It does not mention how incomplete or ambiguous responses were handled.

 It is not mentioned whether participation bias was controlled for.

 The results in Tables 2 and 3 show numbers, but there is no statistical analysis beyond the count.

 The statements made in the results are based on perceptions.

 The discussion in general is too brief and does not relate concepts that one would expect to see, such as adulteration in honey, informality in marketing channels, international trade.

 The differences between countries and their geographical relevance are not explained.

 The conclusions and discussions are not clearly differentiated from each other; likewise, in this section, the reader is not warned that the lack of correlation may be due to the design.

 There are no recommendations, nor are the limitations of the work acknowledged.

Is the study design appropriate and does the work have academic merit?

No

Is the work clearly and accurately presented and does it cite the current literature?

No

If applicable, is the statistical analysis and its interpretation appropriate?

No

Are all the source data underlying the results available to ensure full reproducibility?

No

Are the conclusions drawn adequately supported by the results?

No

Are sufficient details of methods and analysis provided to allow replication by others?

No

Reviewer Expertise:

honey, stingless bees, food biotechnnology

I confirm that I have read this submission and believe that I have an appropriate level of expertise to state that I do not consider it to be of an acceptable scientific standard, for reasons outlined above.

# Response

**Published:** 2025-12-18

**Authors:** Henning Lyngsø FOGED

Thank you for your valuable review of our manuscript.  Your comments are very useful for our amendment of the manuscript. You will in the “Notes to readers” find a general description of the revisions made.      We understand that our original presentation of quantitative analyses, including trade balances for countries represented in responses, could be interpreted as a statistical analysis. We have consequently restructured the manuscript to stress that such analyses are alone done for the purpose of describing the dataset. We also moved these analyses away from the introduction since they are a part of the research and not background material. In this way we intend the manuscript to have a more logical and clear structure. We notice that you suggest the introduction to include an analysis of policies governing the sector. We stress, and have further substantiated how the manuscript is linked to the BeSafeBeeHoney project, which is funded by the EU, wherefore our geographical focus is Europe, where also the vast majority of BeSafeBeeHoney members are located. We do actually mention the main EU policies, including the Honey Directive, the Breakfast Directive, as well as the EU decision of making support to the sector compulsory via the CAP since 1997. We are not entirely clear on the comment that ‘the introduction lacks data relevant to the study, such as policies applied to the beekeeping sector or honey market regulations,’  and would appreciate more specific guidance on which aspects the reviewer considers insufficient or missing! We have noted your comment on “Procedures for ensuring reliability or coding the data are not described.” To our understanding, reliability is of major relevance to consider when analysing data that are produced in a lab or similar, where observations are made, and you want to be ascertained that a solid calibration was ensured. For open-ended questions, we anticipate all respondents provide different and subjective answers, and we don’t want them to calibrate their answers with other respondents since that would lower the quality of the responses. Also, coding is typically something you do for large datasets of the same type as mentioned. However, we did some form of coding for the purpose of describing the policy examples given in the responses, namely in the following ways: 1) country, 2) positive or negative example, 3) the affiliation to the issue of the BeSafeBeeHoney work packages, 4) the countries trade balance, and 5) the policy type.  Likewise, we would like to stress, that in a survey of the nature in question, there are no incomplete answers. There is no defined correct length of the text of an open-ended response. All questions are furthermore voluntary. Regarding the issue of ambiguity, we did not identify any ambiguous responses in our analysis: All responses were clear for us with respect to the mentioned 5 “codings” apart from the issue. There were examples of responses, which we did not understand, such as dealing with renewable energy, but since they were exceptional answers they have not had any effect on conclusions, which point at general patterns in raised policy issues. We would appreciate if you could provide specific examples from the published dataset where ambiguity is perceived that could affect our conclusions, so that we may address them appropriately.  In addition, we emphasise, that by using open-ended questions, we do anticipate that all responses are “biased” and subjective, since there is no correct answer, and you cannot respond to such a survey like this in an objective and impartial way. The prerequisite is that respondents are affiliated in one or another way with the apiculture sector and are willing to express their opinions about the policies that influences the sector.  Once again, we sincerely appreciate your comments. We acknowledge that the original version of the manuscript lacked sufficient clarity and logical structure. We hope that the revised version adequately addresses these issues. Should any concerns remain, we would be grateful for more detailed guidance on the specific points that require further improvement, taking into account the nature of our research.

# Reviewer report: Reviewer response for version 1

**DOI:** 10.21956/openreseurope.22978.r64621

**Published:** 2025-11-27

**Authors:** Małgorzata Dżugan

**Affiliations:** 1 Uniwersytet Rzeszowski, Rzeszów, Poland

The topic addressed in this publication is extremely timely and valuable for the future of the beekeeping market, not only in EU countries. The authors attempted to determine which of the three policies: 1) strategies (implemented or planned), 2) regulations and regulatory standards, and 3) subsidy programs, survey respondents consider crucial for promoting the beekeeping sector. The research was conducted using open web-based survey. The methodology of such research is based on two pillars: a well-prepared, well-thought-out survey questionnaire and a large number of responses (for proper statistical evaluation of observed trends).

  In my opinion, both conditions were not met. The survey design does not ensure reliable responses; it relies on open-ended explanations and requires knowledge of the subject from the respondent. To avoid accidental errors, at least 100 surveys or a consensus position of a beekeeping organization in a given country should have been collected. The low survey response rate (16 in total) absolutely does not allow for correlation of the survey results with the annual trade balance of the honey market per capita. Furthermore, the conclusions should be preceded by a discussion of the results and constitute a summary of the whole publication.

 Therefore, I cannot recommend this work for indexing in its current form. Considering the informative value and importance of the topic for the health of the beekeeping sector and probably element of BeSafeBeeHoney COST project realization, I suggest the authors treat their study as preliminary and submit a Short communication that removes the above mentioned correlations and presents examples of policy implementation for the beekeeping sector in various EU countries. I encourage you to expand the group of survey respondents and include a more comprehensive explanation of the policy for the apiculture sector in the online questionnaires, e.g. in the form of a short video tutorial.

Is the study design appropriate and does the work have academic merit?

Partly

Is the work clearly and accurately presented and does it cite the current literature?

Yes

If applicable, is the statistical analysis and its interpretation appropriate?

No

Are all the source data underlying the results available to ensure full reproducibility?

Partly

Are the conclusions drawn adequately supported by the results?

No

Are sufficient details of methods and analysis provided to allow replication by others?

Yes

Reviewer Expertise:

Honey and other bee product analysis - quality evaluation and using in apitherapy

I confirm that I have read this submission and believe that I have an appropriate level of expertise to state that I do not consider it to be of an acceptable scientific standard, for reasons outlined above.

# Response

**Published:** 2025-12-18

**Authors:** Henning Lyngsø FOGED

Thank you for your valuable review of our manuscript.

 Your comments are very useful for our amendment of the manuscript. You will in the “Notes to readers” find a general description of the revisions made.   In addition, we regret that our manuscript was not sufficiently clear and appreciate the opportunity to clarify, that our research did not intend to determine which of three policies respondents consider crucial for promoting the beekeeping sector. We have in various ways reformulated the manuscript to more clearly describe the objective of the research, especially in the formulation of the objectives. We are of the opinion, that using open ended questions for exploratory purposes does not require more than 100 responses, since it is not intended or even suited for statistical analysis, and we can assure that the design of the questionnaire was thoroughly considered. The methodological section emphasises now more clearly, also by use of a reference, that the use of open-ended questions is a recognised social-science method for exploring perceptions, that these are not meant for statistical analyses, but for qualitative analyses, and that each response is valuable and unique.  We understand that our original presentation of quantitative analyses, including trade balances for countries represented in responses, could be interpreted as a statistical analysis. We have consequently restructured the manuscript to stress that such analyses are alone done for the purpose of describing the dataset. We also moved these analyses away from the introduction since they are a part of the research and not background material. In this way we intend the manuscript to have a more logical and clear structure. 

 Once again, we do appreciate you review comments. We acknowledge that the original version was of insufficient quality and not entirely clear and logically structured. We hope that the revised version adequately addresses your concerns. If any issues remain, we would appreciate further clarification of your specific concerns, given the scope and nature of our research.

# Reviewer report: Reviewer response for version 1

**DOI:** 10.21956/openreseurope.22978.r61937

**Published:** 2025-10-14

**Authors:** Ester Noguer-Juncà

**Affiliations:** 1 University of Lleida, Lleida, Spain

Dear Authors,

 Thank you for the opportunity to review this work. While your paper offers valuable insights, several critical issues remain unaddressed.

 The originality of the research is not sufficiently established, particularly regarding its contribution to the existing literature and the research gap it aims to address.

 The Introduction section is well-developed. Perhaps the explanation of BeSafeBeeHoney Project could be detailed.

 One of the major weakness of the paper, in my view, lies in its methodology section. The number of surveys is insufficient to generalize the findings. It is necessary to add more information about the processes taken to ensure validity, reliability, and credibility of the results. 

 The Results section is well-developed, but the information could be detailed. 

 In my view, the Discussion and conclusion section represents another weak point of the paper.

 It is necessary to create two separate sections. There is no connection between the discussion and the background. 

 The paper also lacks an explanation of its theoretical and practical contributions, as well as a discussion of its limitations and suggestions for future research directions.

 The quality of communication and the use of English are adequate.

 Given these limitations, I regret to inform you that I reject the manuscript in its current form.

Is the study design appropriate and does the work have academic merit?

Partly

Is the work clearly and accurately presented and does it cite the current literature?

Yes

If applicable, is the statistical analysis and its interpretation appropriate?

No

Are all the source data underlying the results available to ensure full reproducibility?

Partly

Are the conclusions drawn adequately supported by the results?

Partly

Are sufficient details of methods and analysis provided to allow replication by others?

No

Reviewer Expertise:

food tourism, food marketing, primary sector and tourism, tourism and gender

I confirm that I have read this submission and believe that I have an appropriate level of expertise to state that I do not consider it to be of an acceptable scientific standard, for reasons outlined above.

# Response

**Published:** 2025-12-18

**Authors:** Henning Lyngsø FOGED

Thank you for your valuable review of our manuscript.  Your comments are very useful for our amendment of the manuscript. You will in the “Notes to readers” find a general description of the revisions made. 

 In addition, we acknowledge your concern that the original presentation might be interpreted as implying a statistical analysis of the survey responses, alike methods used in for instance biological research disciplines. We have in several ways amended the manuscript to clarify that this is not the intention: The methodological section emphasise more clear, that the data is collected by use of open-ended questions, which is a recognised social-science method for exploring perceptions, that these are not meant for statistical analyses, but for qualitative analyses, and that each response is valuable and unique. It is likewise stressed in the results section, that the three issues that were perceived as being most decisive for a thriving apiculture, were in fact dealt with in most of the responses, wherefore we do not feel that having additional responses would add substantial to the validity or representation of the conclusions. Also, it is now clearer that various quantification of the survey responses are alone having the purpose of describing the dataset. We hope that the amended manuscript cannot any more cause misunderstandings about the social science nature of our research and the use of open-ended questions for explorative purposes.  Once again, we do appreciate you review comments. We acknowledge that the original version was of insufficient quality and not entirely clear and logically structured. However, we do hope that you are able to approve the revised version, and that you otherwise will explain in more detail what concerns you have, given the nature of our research.
